# Pancreatic perfusion and its response to glucose as measured by simultaneous PET/MRI

**DOI:** 10.1007/s00592-019-01353-2

**Published:** 2019-04-26

**Authors:** Daniel Espes, Elin Manell, Anneli Rydén, Lina Carlbom, Jan Weis, Marianne Jensen-Waern, Leif Jansson, Olof Eriksson

**Affiliations:** 1 Department of Medical Cell Biology, Uppsala University, 751 23 Uppsala, Sweden; 2 Department of Medical Sciences, Uppsala University, 751 83 Uppsala, Sweden; 3 Department of Clinical Sciences, Swedish University of Agricultural Sciences, Uppsala, Sweden; 4 Department of Surgical Sciences, Uppsala University, 751 83 Uppsala, Sweden; 5 Department of Medical Physics, Uppsala University Hospital, 751 83 Uppsala, Sweden; 6 Science for Life Laboratory, Department of Medicinal Chemistry, Uppsala University, Dag Hammarskjölds väg 14C, 3tr, 751 83 Uppsala, Sweden

**Keywords:** Pancreas perfusion, PET/MRI scanner, Hybrid scanner, Diabetes

## Abstract

**Aims:**

Perfusion of the pancreas and the islets of Langerhans is sensitive to physiological stimuli and is dysregulated in metabolic disease. Pancreatic perfusion can be assessed by both positron emission tomography (PET) and magnetic resonance imaging (MRI), but the methods have not been directly compared or benchmarked against the gold-standard microsphere technique.

**Methods:**

Pigs (*n* = 4) were examined by [^15^O]H_2_O PET and intravoxel incoherent motion (IVIM) MRI technique simultaneously using a hybrid PET/MRI scanner. The pancreatic perfusion was measured both at basal conditions and after intravenous (IV) administration of up to 0.5 g/kg glucose.

**Results:**

Pancreatic perfusion increased by 35%, 157%, and 29% after IV 0.5 g/kg glucose compared to during basal conditions, as assessed by [^15^O]H_2_O PET, IVIM MRI, and microspheres, respectively. There was a correlation between pancreatic perfusion as assessed by [^15^O]H_2_O PET and IVIM MRI (*r* = 0.81, *R*^2^ = 0.65, *p* < 0.01). The absolute quantification of pancreatic perfusion (ml/min/g) by [^15^O]H_2_O PET was within a 15% error of margin of the microsphere technique.

**Conclusion:**

Pancreatic perfusion by [^15^O]H_2_O PET was in agreement with the microsphere technique assessment. The IVIM MRI method has the potential to replace [^15^O]H_2_O PET if the pancreatic perfusion is sufficiently large, but not when absolute quantitation is required.

## Introduction

Between 10 and 20% of the total pancreatic blood flow in animals is diverted through the islets of Langerhans during basal physiological conditions, even though they only constitute 1–2% of the total pancreatic volume [1, 2]. In addition, the islet blood flow increases 2–3 times in response to glucose [3]. Islet blood flow is independently regulated from that of the exocrine blood flow. Moreover, the mechanism for glucose regulation of the islet blood flow is still not completely understood, but it is likely dependent on a combination of local and systemic processes, e.g., vasodilators and constrictors as well as the autonomic nervous system [4, 5].

Sufficient pancreatic and islet blood flow at basal conditions and in response to metabolic challenges is critical for ensuring oxygen delivery and to adequately monitor the blood glucose concentrations. The blood flow is also of importance for a rapid hormonal response from the islets of Langerhans and the secretion of hormones into the circulation.

Preclinical assessment of pancreatic perfusion is usually performed by the microsphere technique [2], which is considered as the gold standard but is also terminal and not applicable in the clinical setting. Recently, the positron emission tomography (PET) radiopharmaceutical [^15^O]H_2_O (Oxygen-15 isotopically labeled water) has enabled quantitative assessments of pancreatic perfusion also in humans [6].

Using this method, it was shown that an oral glucose challenge potentiated the pancreatic perfusion by up to 50% in healthy humans [6]. Furthermore, both the basal- and glucose-stimulated pancreatic perfusion were aberrantly regulated in individuals with obesity, type 1 diabetes (T1D), and type 2 diabetes (T2D) [6–10], but could be normalized by intervention (such as bariatric surgery [11]).

Clearly, both the preclinical and the clinical literature point to an important role of pancreatic perfusion defects in metabolic disease. The [^15^O]H_2_O PET technique has high precision and is quantitative. However, the requirement of an expensive radioactive contrast agent impedes its application in large-scale interventional clinical studies.

Recent developments in hybrid PET and magnetic resonance imaging (MRI) scanners enable the assessment of pancreatic perfusion by [^15^O]H_2_O PET, simultaneously with non-contrast agent-dependent diffusion-weighted imaging (DWI) MRI technique.

The purpose of the current study was to directly compare MRI with [^15^O]H_2_O PET and the gold-standard microsphere technique for the assessment of pancreatic perfusion in pigs.

## Method and materials

### Animal model

The study was approved by the Ethical Committee for Animal Research of the Uppsala Region and was performed according to the Uppsala University guidelines on animal experimentation (UFV 2007/724). High-health herd-certified normoglycemic male pigs (Yorkshire × Swedish Landrace x Hampshire, male, *n* = 4, weight 28.5–35 kg) were used. Anesthesia was induced by an IM injection of tiletamine–zolazepam (Zoletil Forte^®^ vet. 250 mg/mL) 5 mg/kg and medetomidine (Domitor^®^vet. 1 mg/mL) 0.025 mg/kg and maintained by an IV infusion of ketamine (Ketamin^®^ 10 mg/mL) 28 mg/kg × h, midazolam (Midazolam Actavis^®^ 5 mg/mL) 0.1 mg/kg × h, and fentanyl (Fentanyl B. Braun^®^ 50 µg/mL) 3.5 µg/kg × h. A detailed description of the MRI compatible pig anesthesia procedure was previously published [12]. Venous catheters were placed in the left and right auricular vein for anesthesia infusion (left ear) or [^15^O]H_2_O and glucose infusion (right ear). In one pig, an arterial Seldinger catheter was placed in the arteria carotis with the tip placed in the left cardiac ventricle for administration of microspheres. Additionally, an arterial catheter was also placed in the arteria femoralis to obtain a reference sample.

### PET/MRI perfusion examinations and glucose challenges

Pigs were examined up to three times during 1 day using a PET/MRI scanner (Signa, GE Healthcare). The instrument is equipped with a 3 T magnet and allows for simultaneous PET/MRI measurements.

Pancreas perfusion was assessed simultaneously and independently by [^15^O]H_2_O PET and DWI, either at basal conditions or after IV administration of glucose. In one pig, perfusion was also assessed by injection of microspheres. Table 1 demonstrates the details on the experimental procedures.

**Table 1:**
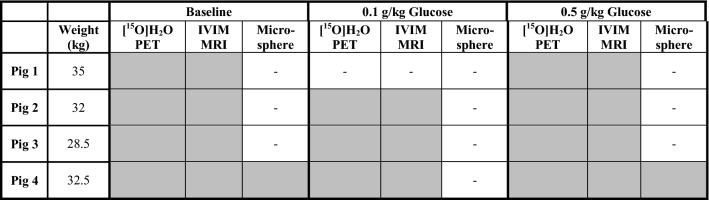
Procedures performed at each experiment

Gray boxes indicate performed examinations, and white boxes indicate non-performed procedures

The first PET/MRI examination aimed to measure the pancreatic perfusion at basal conditions. Before subsequent PET/MRI examinations, the pigs were given an IV glucose challenge (0.1 g/kg or 0.5 g/kg). Glucose was administered as a 2-min infusion of 10 or 50 mL solution (300 mg/mL), immediately before the PET/MRI examination. Blood glucose was sampled before and after each PET/MRI examination.

One pig was administered colored microspheres to measure the pancreatic and islet perfusion [2]. In this pig, 12.1 mL of a microsphere solution (approximately 10^7^ red microspheres, 15.5 ± 0.3 µm, EZ Trac, Los Angeles, CA, USA) was administered intra-arterially in the left cardiac ventricle 1 min after the start of the PET/MRI examination during basal conditions. An arterial reference sample was withdrawn from the femoral catheter, starting 5 s before the microsphere injection and continuing for 60 s.

The same procedure was repeated during the 0.5 g/kg glucose challenge and PET/MRI examination, with the exception that yellow microspheres were administered instead.

After the final PET/MRI examination, the pig was euthanized. Biopsies (1–2 g each) were taken from the different regions of the pancreas (six biopsies) as well as the right and left kidney cortex. The arterial samples and tissue biopsies were then frozen at − 20 °C until their microsphere contents were determined [2].

### [^15^O]H_2_O perfusion assessment by PET

Each pig was positioned with the pancreas in the center of the scanner. 194 ± 46 MBq [^15^O]H_2_O (corresponding to 6.1 ± 1.2 MBq/kg) was IV administered by a contrast pump (Medrad Stellant, Bayer, Whippany, NJ, USA) (0.8 mL/s for 10 s), followed by flushing with 15 ml NaCl (1 mL/s for 15 s). A dynamic PET sequence over 10 min was started simultaneously with the activation of the infusion pump.

The [^15^O]H_2_O PET datasets were reconstructed into 26 frames, according to the following frame sequence: 1 × 10, 8 × 5, 4 × 10, 2 × 15, 3 × 20, 2 × 30, and 6 × 60 s.

Images were reconstructed using the VPFX algorithm (5 mm post-filter, diameter 50 cm). Vendor default MRI scan was used for attenuation correction.

[^15^O]H_2_O PET images were analyzed in PMOD 3.7 (PMOD Technologies, Zurich, Switzerland). Regions of interests (ROIs) were segmented over the pancreas, spleen, and kidney cortex on transaxial co-registered MRI images. The ROIs were summed into volumes of interest (VOIs) and overlaid on transaxial PET SUV-corrected images. In the descending aorta, single voxels fully within the vessel lumen (to minimize the partial volume effects) were segmented directly on early PET SUV-corrected images.

For each PET examination, perfusion (expressed as mL/min/g tissue) was calculated by the 1-tissue compartment (1TC) model for [^15^O]H_2_O (PKIN module, PMOD 3.7), based on the dynamic [^15^O]H_2_O uptake in the pancreas, spleen, and kidney cortex using the aortic signal as the input function. Parametric perfusion images were generated by the PMOD pixel-wise modeling module using the [^15^O]H_2_O 1TC, according to Alpert (PXMod, PMOD 3.7).

### Perfusion assessment by MRI

The intravoxel incoherent motion (IVIM) technique was used for perfusion fraction f assessment [13]. IVIM refers to the movement of water molecules due to diffusion and capillary perfusion. Dimensionless perfusion fraction f represents the fraction of a voxel volume occupied by the capillaries. The technique is based on the separation of true diffusion (*D*) from perfusion related “pseudo-diffusion” (D*) using a diffusion-weighted sequence with multiple *b*-values. Fat-suppressed and respiratory-triggered DW images were acquired by a single-shot spin-echo sequence with echo-planar read-out. The following parameters were used: TE 70 ms, FOV 350 × 350 mm^2^, spatial resolution 1.4 × 1.4 × 6 mm^3^, interslice gap 0 mm, bandwidth per pixel (BW) 976 Hz, and number of scans 3. Diffusion-encoding gradients (*b* = 0, 20, 75, 150, 450, and 900 s/mm^2^) were sequentially applied along the three orthogonal directions. The maps of (isotropic) apparent diffusion coefficient (ADC) were automatically calculated by the scanner with mono-exponential fitting.

The same pancreas, spleen, and kidney cortex VOIs, as used for the PET analysis, were transferred to the DWI images. Additionally, a VOI outside of the pig was used to generate the background signal. The background values were subtracted from the VOI values for each *b*-value in each scan. Then, the output was plotted against the *B*-values and the resulting curve fitted to the bi-exponential equation given below, where *x* = *b*-values, *f* = capillary volume fraction (or fraction of fast diffusion), *D* = true diffusion (mm^2^/s), and *P* = fast diffusion (perfusion related diffusion) (mm^2^/s) using the software package GraphPad 6.0 (GraphPad Software, La Jolla, CA, USA).

$$Y\left( x \right) = \left( {1 - f} \right)*{\text{e}}^{ - x*D} + f*{\text{e}}^{{ - x*\left( {D + P} \right)}}$$

### Microsphere perfusion assessment

The number of microspheres in the pancreas, islets, kidney cortex, and arterial reference sample was calculated by manual counting. For this purpose, an inverted microscope equipped with both bright- and dark-field illumination was used. More than 2000 microspheres were counted in each sample. The blood flow could then be calculated, according to the formula *Q*_org_ = *Q*_ref _× *N*_org_/*N*_ref_, where *Q*_org_ is the organ blood flow (ml/min), *Q*_ref_ is the withdrawal rate of the reference sample, *N*_org_ is the number of microspheres present in the organ, and *N*_ref_ is the number of microspheres in the reference sample [2].

### Statistical analysis

The results are given as mean ± SEM. Differences between the groups were assessed by two-tailed, paired *t* test, using a confidence level of 0.95 using GraphPad 6.0 (GraphPad Software, La Jolla, CA, USA). Correlation was assessed by the Pearson correlation coefficient.

## Results

### Experimental outcome

Experimental procedures were performed as planned, except for in pig 1, where only one glucose challenge (0.5 g/kg) could be performed due to a delay in the [^15^O]H_2_O production (Table 1). All pigs were normoglycemic (4.1–5.7 mM) at baseline. Administration of 0.5 g/kg glucose by IV increased the B-glucose to 10–15 mM (mean 12.6 mM, range 7.6–16.3 mM), while IV administration of 0.1 g/kg glucose increased the B-glucose more modestly (mean 6.3 mM, range 3.3–9.9 mM).

### Pancreatic perfusion measurement by PET

**Fig. 1:**
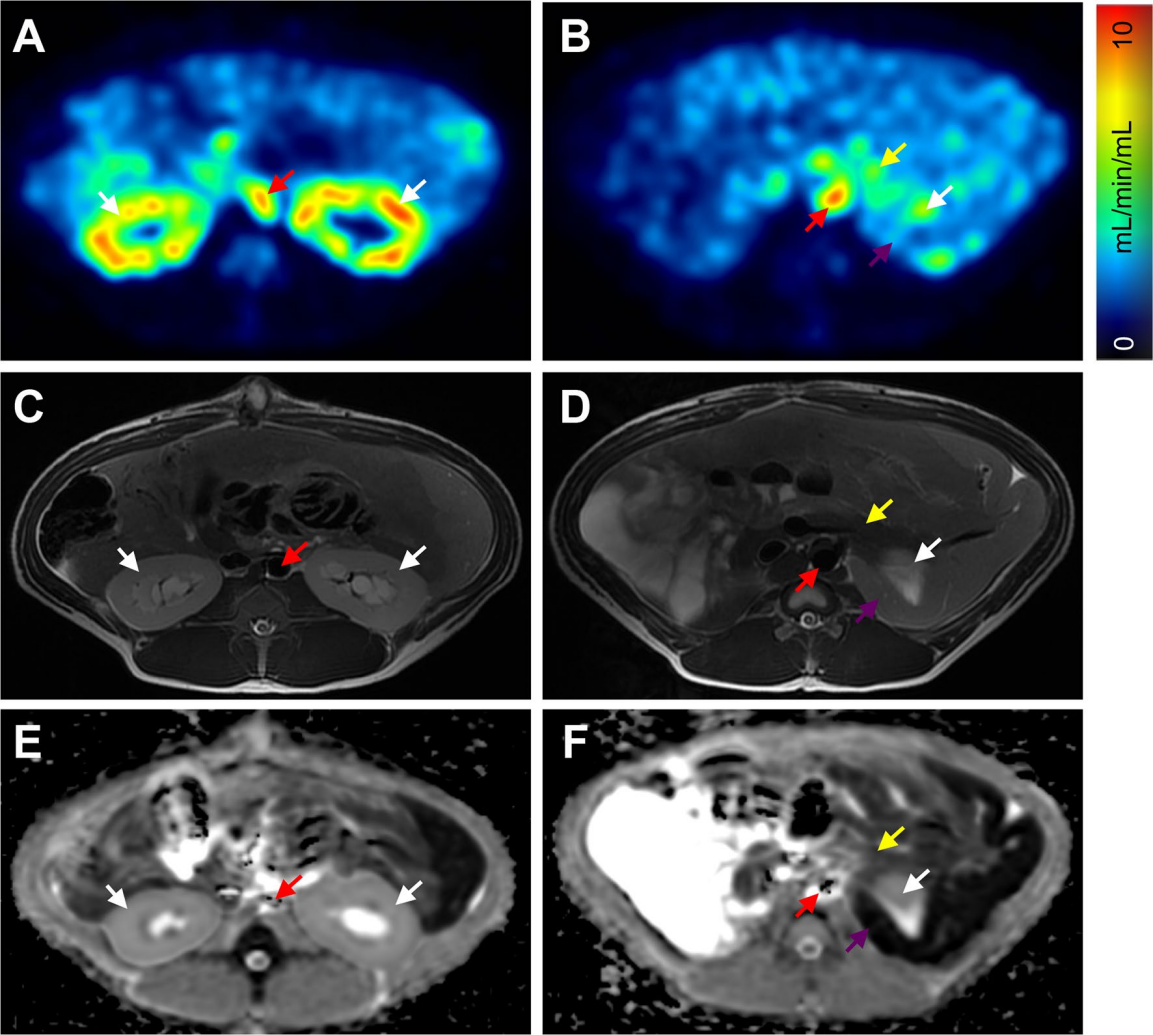
Representative parametric [^15^O]H_2_O PET blood flow images (**a**, **b**), corresponding anatomical MRI images (**c**, **d**), and ADC MRI maps (**e**, **f**) from the baseline examination of pig 4

**Fig. 2:**
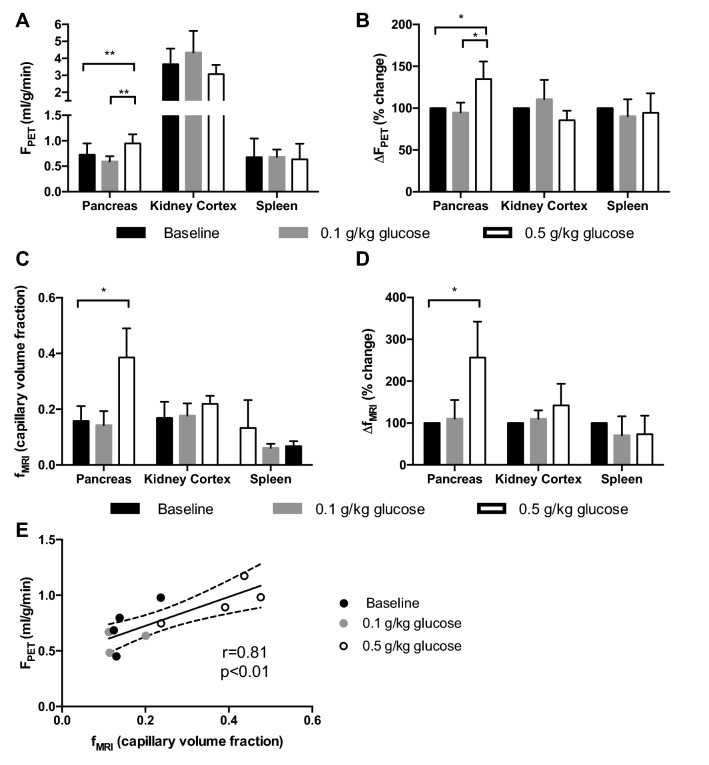
Perfusion and change in perfusion in abdominal tissues before and after the glucose challenge, as assessed by [^15^O]H_2_O PET (**a**, **b**) and IVIM MRI (**c**, **d**). There was a correlation between the pancreatic perfusion at different degrees of stimulation, as assessed by [^15^O]H_2_O PET and IVIM MRI (**e**)

Pancreas and kidney cortex exhibited strong perfusion on PET parametric blood flow maps of the abdomen (Fig. 1a, b). Representative corresponding anatomical MRI (Fig. 1c, d) and ADC maps (Fig. 1e, f) from the same subject are also shown. Pancreatic perfusion during basal conditions was 0.73 ± 0.22 mL/min/g as assessed by [^15^O]H_2_O PET (Fig. 2a). Moreover, 0.5 g/kg of glucose, administered by IV, increased the perfusion in the pancreas (0.95 ± 0.18 mL/min/g, *p* < 0.01) but not in the kidney cortex or the spleen. This corresponded to an average increase in the pancreatic perfusion by 35% (*p* < 0.05) (Fig. 2b). Thereafter, IV administration of 0.1 g/kg of glucose did not affect the pancreatic perfusion appreciably.

### Pancreatic perfusion measurement by MRI

Capillary volume fraction (*f*), as measured by DWI-MRI and used as an index of perfusion, was 0.16 ± 0.05 in the pancreas (Fig. 2c). Administration of 0.5 g/kg of glucose by IV caused an increase in the pancreatic signal to 0.39 ± 0.10, corresponding to an average increase of 157% (Fig. 2d). No change was seen in the perfusion as measured by DWI-MRI, after the IV administration of 0.1 g/kg of glucose. The IV glucose challenge did not affect perfusion in the kidney cortex or the spleen as assessed by DWI-MRI. Pancreatic perfusion as measured by perfusion fraction f correlated with the assessment with [^15^O]H_2_O PET (*r* = 0.81, *R*^2^ = 0.65, *p* < 0.01) (Fig. 2e).

### Pancreatic perfusion measurement by microspheres

The blood flow value in the right and the left kidney cortex of pig 4 was in the range of 10%, verifying an adequate mixing of the microspheres with the arterial circulation. The blood flow in the exocrine pancreas as assessed by the microsphere technique (an average of six biopsies) was 0.7 ml/min/g at baseline, which increased by 29% to 0.9 ml/min/g after IV administration of 0.5 g/kg of glucose. In the same animal, the baseline perfusion assessed by [^15^O]H_2_O PET was 0.8 ml/min/g, which increased by 23% to 0.98 ml/min/g after IV administration of 0.5 g/kg glucose. The islet specific blood flow, as determined by the microspheres, was 52 µl/min/g pancreas at baseline and 78 µl/min/g pancreas (an increase of 50%) after IV administration of 0.5 g/kg of glucose.

## Discussion

The microsphere technique has been considered the gold-standard preclinical technique for assessing blood flow for decades. Separately, [^15^O]H_2_O PET has emerged as a fully quantitative technique, perfectly mimicking water flow in human tissues. Myocardial perfusion imaging agents, including [^15^O]H_2_O, have been validated against microspheres in large animal models [14, 15], but so far no similar direct comparison has been made with regard to pancreatic perfusion.

Here, we show that the pancreatic perfusion, measured by [^15^O]H_2_O PET, closely resembles (within 15%) values measured by the microsphere technique, both the absolute quantification and the measurement of change.

The major drawback with [^15^O]H_2_O PET is the logistics of the examination, making large-scale clinical deployment difficult. The examination requires radiosynthesis of [^15^O]H_2_O from a cyclotron located in close proximity to the PET scanner due to the 2 min ^15^O half-life. The combination of logistics and state-of-the-art technology is usually only available at major imaging centers, with heavy focus on research.

A noninvasive, contrast agent-free examination retaining the precision and sensitivity of the [^15^O]H_2_O methodology would greatly assist in disseminating pancreatic perfusion imaging.

Here, we examined the correlation of pancreatic perfusion assessed simultaneously by the [^15^O]H_2_O PET and MRI (IVIM) technique. Another possibility to quantify perfusion with MRI is dynamic contrast-enhanced (DCE). It has, for example, been used to assess perfusion both in native pancreas in response to a glucose challenge [16] and in the setting of islets transplantation [17]. However, DCE-MRI requires the intravenous administration of a contrast agent (often gadolinium-based). Therefore, the noninvasive IVIM MRI technique was used in this comparison.

Capillary volume fraction (*f*) was sensitive to the perfusion change and correlated to the [^15^O]H_2_O PET measurement. Additionally, the percentage increase in perfusion fraction after glucose infusion was three times larger when assessed with IVIM than with [^15^O]H_2_O PET. The IVIM MRI method therefore possesses the potential to replace the [^15^O]H_2_O PET to determine a change in the pancreatic perfusion, but not if absolute quantitation is required. This is due to the fact that the [^15^O]H_2_O PET measures the perfusion in physically relevant units (ml perfusion per ml tissue per minute), while IVIM outcome is measured by the less intuitive capillary volumes fraction unit.

The response in pancreatic perfusion was consistently noted after infusion of 0.5 g/kg of glucose. The increase in the whole pancreas perfusion, based on the [^15^O]H_2_O PET assessment, was on average 35%. The majority of the glucose-potentiated perfusion increase is localized in the pancreatic islets. Our microsphere assessment showed a 50% increase in islet perfusion. Although impressive, this can only partially account for the whole pancreas perfusion increase (35%), since the islets only constitute 1–2% of the pancreatic volume.

The inter-study reproducibility of [^15^O]H_2_O PET and IVIM in the pancreas is difficult to assess due to the limited literature on imaging studies of perfusion in pig pancreas. However, our group has performed similar studies, which can be used as comparative material. The basal pancreatic perfusion was previously assessed by [^15^O]H_2_O PET/CT in healthy and streptozotocin (STZ)-induced diabetes in pigs of similar weight and age as in the current study [18]. In the study by Nalin et al., healthy non-diabetic pigs (*n* = 4) exhibited a basal pancreatic perfusion of 0.45 ± 0.09 ml/min/g. This is lower compared to the basal pancreatic perfusion measured in the current study (0.73 ± 0.22 ml/min/g, n.s.), but this may be due to the differences in the scanner performance (Discovery ST PET/CT vs. Signa PET/MRI) as well as the age of the pigs.

IVIM assessment of the pancreas in pigs during basal conditions was also performed previously in the Signa PET/MRI scanner [12]. The capillary volume fraction (0.18 ± 0.06, *n* = 5) was very similar to the current study (0.16 ± 0.05, *n* = 4, n.s.). Thus, when using animals of similar age in the same scanner, the inter-study reproducibility of the pancreas IVIM assessment seems high.

Pooling the [^15^O]H_2_O PET data from the current and the previous study, healthy (*n* = 8) and STZ-diabetic pigs (*n* = 3) have basal pancreatic perfusion of 0.59 ± 0.21 ml/min/g and 0.24 ± 0.05 ml/min/g (*p* < 0.05), respectively. Thus, beta cell loss is associated with a drastic decrease in the basal pancreatic perfusion. A similar decrease, but lower in magnitude, was observed also in humans [6]. However, in humans, it is difficult to draw conclusions regarding islet blood flow since the perfusion of the entire splanchnic region, both basal and glucose potentiated, is affected in T1D [6]. Thus, it is possible that endothelial dysfunction in the splanchnic region also contributes to the observations in diabetic pigs. Glucose-potentiated [^15^O]H_2_O PET measurements alone, therefore, are unlikely to provide a reliable surrogate marker for pancreatic islet function.

Perfusion imaging techniques have found widespread use in many clinical diagnostic settings. Myocardial perfusion imaging, for example, has gone from a small-scale research tool to a routine examination in cardiovascular disease in the last few decades [19]. Furthermore, myocardial perfusion reserve (MPR) has been shown to be a biomarker for predicting cardiac mortality [20], making it an important endpoint in early phase drug development studies in cardiovascular/metabolic disease.

However, the clinical application of sensitive perfusion imaging techniques, such as PET and MRI, specifically in the pancreas is still in the early research stage, mainly being employed in single-center cross-sectional or longitudinal studies. This is likely due to the lack of validation studies in the pancreas between different imaging techniques and in relation to invasive gold-standard methods, thus, the need for the present study.

Human pancreatic perfusion is dysregulated in metabolic disease [6–10], but importantly, there is evidence that these changes can be normalized or reversed by intervention such as bariatric surgery [10]. Thus, precise assessment of changes in pancreatic perfusion may potentially become a clinical endpoint in mechanistic drug development studies, potentially forming a biomarker for improved pancreatic metabolism. The results here support the notion that both [^15^O]H_2_O PET and IVIM MRI measure pancreas perfusion in accordance with the gold-standard invasive techniques and thus are precise markers suitable as endpoints in clinical studies of the pancreas.

## Conclusion

Pancreatic perfusion by [^15^O]H_2_O PET was in agreement with the microsphere technique assessment. The IVIM MRI method has the potential to replace the [^15^O]H_2_O PET if the pancreatic perfusion is sufficiently large, but not when absolute quantitation is required.
